# Effects of bevacizumab on endoplasmic reticulum stress in hypoxic retinal pigment epithelial cells

**DOI:** 10.1371/journal.pone.0179048

**Published:** 2017-06-07

**Authors:** Joo-Hee Park, Moosang Kim, Jong-Hyun Oh

**Affiliations:** 1 Department of Ophthalmology, Dongguk University Ilsan Hospital, Goyang, South Korea; 2 Department of Ophthalmology, School of Medicine, Kangwon National University, Chuncheon, South Korea; Duke University School of Medicine, UNITED STATES

## Abstract

**Purpose:**

To investigate the effects of bevacizumab on endoplasmic reticulum (ER) stress in human retinal pigment epithelial (RPE) cells cultured under hypoxic conditions.

**Methods:**

RPE cells (ARPE–19) were cultured under hypoxic conditions (1% O_2_) with or without bevacizumab (0.3125 mg/mL) for 24 and 48 h. Cell viability was measured by a PrestoBlue assay. The expression of vascular endothelial growth factor (VEGF), binding protein/glucose-regulated protein 78 (BiP/GRP78), and C/EBP homologous protein-10 (CHOP) mRNA was measured by quantitative real-time polymerase chain reaction (qRT-PCR). BiP/GRP78 and CHOP protein levels in the cells were assessed by western blot. VEGF protein in the media was quantified by enzyme-linked immunosorbent assay (ELISA).

**Results:**

Under hypoxic conditions, cell viability decreased and mRNA and protein levels of VEGF, BiP/GRP78, and CHOP increased compared to those under normoxic conditions. Bevacizumab improved cell viability and reduced the expression of VEGF mRNA under hypoxic conditions. Bevacizumab also reduced the expression of both mRNA and protein of two ER stress indicators, BiP/GRP78 and CHOP, under hypoxic conditions.

**Conclusions:**

Bevacizumab mitigated ER stress in human RPE cells cultured under hypoxic conditions. This effect may be involved in the improved cell viability and reduction of VEGF expression after bevacizumab treatment of hypoxic RPE cells *in vitro*. However, the effects of bevacizumab on RPE cells under experimental conditions are unlikely to be clinically equivalent to those in the human eye.

## Introduction

Age-related macular degeneration (AMD) is an important cause of blindness in the elderly [1]. The disease is divided into a dry (atrophic) type and a wet (neovascular) type [2]. The advanced form of dry AMD is called geographic atrophy (GA) and is characterized by the loss of retinal pigment epithelium (RPE) and photoreceptor cells [1, 3]. Wet AMD is characterized by choroidal neovascularization (CNV) [1, 3]. AMD is a multifactorial disease, and the pathogenic mechanism of AMD is complex. In one possible hypothesis, deposits between the RPE layer and choriocapillaries, known as drusen, may prevent the diffusion of oxygen and nutrients from the choriocapillaries to the RPE monolayer and photoreceptors [4]. As a consequence, the RPE gradually degenerates and GA occurs [5]. In wet AMD cases, cellular hypoxia may result in the overexpression of angiogenic growth factors such as vascular endothelial growth factor (VEGF), which, in turn, induces neovascularization from the choriocapillaries [6]. There is no well-recognized treatment for halting the progression of early dry AMD to GA. In contrast, the treatment of CNV is well established and recently, anti-VEGF drugs such as bevacizumab have been used effectively for patients with wet AMD [7].

The endoplasmic reticulum (ER) is a major intracellular organelle responsible for protein and lipid biosynthesis, protein folding and trafficking, and calcium homeostasis [8]. The accumulation of unfolded or misfolded proteins in the ER, a condition known as ER stress, is induced by various pathological conditions, including oxidative stress, glucose deprivation, disruption of calcium homeostasis, and viral infection [9–11]. ER stress generates a conserved response termed the unfolded protein response (UPR). The UPR is a sophisticated cellular signaling pathway that restores cellular homeostasis, but excessively strong or prolonged ER stress can eventually lead to cell death [12, 13]. Binding protein/glucose-regulated protein 78 (BiP/GRP78) and C/EBP homologous protein-10 (CHOP) are the two main proteins involved in ER stress and the UPR and the increased levels of these two proteins is an indicator of ER stress. BiP/GRP78 is a molecular chaperone that is considered a marker for ER stress induction, whereas the activation of CHOP is closely linked to apoptosis [11, 14].

It has been suggested that ER stress is involved in the pathogenesis of AMD [8, 15].

Cigarette smoking is a well-known environmental risk factor for AMD [16]. ER stress and the UPR are involved in RPE cell apoptosis induced by cigarette smoke-related oxidative injury [17]. In addition, ER stress may be related to CNV formation by upregulating VEGF expression and promoting angiogenesis [18, 19]. In the present study, we aimed to investigate the effects of bevacizumab, an anti-VEGF drug used for wet AMD, on ER stress in human RPE cells cultured under hypoxic conditions.

## Materials and methods

### Cell culture

Human RPE cell line (ARPE-19) was purchased from American Type Culture Collection (ATCC; Manassas, VA, USA). Cells were maintained in Dulbecco’s modified Eagle’s medium/F-12 nutrient medium (DMEM/F-12; Gibco BRL, Carlsbad, CA, USA) supplemented with 10% heat-inactivated fetal bovine serum (Gibco BRL) and 1% penicillin-streptomycin. The cells were plated in 185 cm^2^ tissue flasks and cultured in a humidified incubator at 37°C in an atmosphere of 5% CO_2_. RPE cells within the first 10 passages were selected and placed into appropriate culture plates for the experiments. Semiconfluent cultures (70–80% confluency) were serum-starved for 24 h. The cells were additionally cultured in different oxygen conditions with or without bevacizumab treatment, as indicated below. The culturing in different conditions was conducted in a single day and three or more experiments were independently performed on different days.

### Hypoxia and drug treatment

In the preliminary experiments, RPE cells were incubated in 3% O_2_, 5% CO_2_, and 92% N_2_ atmosphere and in 1% O_2_, 5% CO_2_, and 94% N_2_ atmosphere, using an O_2_ incubator (Biofree, Seoul, Korea), for 24 h and 48 h. Cell viability was compared with that in the control cells, which were cultured in a humidified incubator with 21% O_2_, 5% CO_2_, and 74% N_2_ atmosphere.

In the hypoxic group, RPE cells were incubated in 1% O_2_, 5% CO_2_ and 94% N_2_ atmosphere using an O_2_ incubator, for 24 h and 48 h, whereas in the control group, RPE cells were cultured in a humidified incubator with 21% O_2_, 5% CO_2_, and 74% N_2_ atmosphere.

Bevacizumab (Avastin^TM^; Genentech, Inc., San Francisco, CA, USA) at a final concentration of 0.3125 mg/mL, which was roughly equal to the concentration used clinically [20], was used to treat RPE cells during exposure to hypoxic condisions (hypoxia+bevacizumab group). In addition, RPE cells were treated with various concentrations of bevacizumab (0.0195, 0.0391, 0.0781, 0.3125, and 0.6250 mg/mL) during exposure to hypoxic conditions to assess cell viability.

### PrestoBlue assay

Cell viability was measured using PrestoBlue (PB) reagent (Invitrogen, Carlsbad, CA, USA) according to the manufacturer’s protocol. Briefly, a mixture of 10% PB was added to cells in the culture media. The absorbance was read at 570 nm after RPE cells were incubated for 2 h with PB reagent. Each sample was measured three times. Each experiment was repeated at least three times and two or three samples were obtained from each independent experiment.

### Quantitative real-time PCR

Total RNA was isolated from the cultured cells by the Trizol (Invitrogen) method and the purity and yield of the RNA were determined spectrophotometrically. One microgram of total RNA from each sample was reverse transcribed into cDNA, using PrimeScript^TM^ RT Master Mix (Takara Bio Inc., Otsu, Shiga, Japan) in a total volume of 20 μL, according to the manufacturer’s instructions. Five hundred nanograms of cDNA were used as a template on a Light Cycler 480 (Roche, Mannheim, Germany), using the SYBR Green Premix EX Taq kit (Takara Bio Inc.). The primer sequences used in this study are shown in Table 1. Dissociation curves were generated to check for the specificity of primer annealing to the template. The expression level of each target gene was calculated by the comparative threshold cycle method (2^–ΔΔCt^) with GAPDH as the control gene. Each experiment was repeated at least three times and two or three samples were obtained from each independent experiment.

**Table 1:** The sequences of target genes in this study.

Gene	GenBank No.	Sequence (5’– 3’)	
BiP/GRP78	NM005347	F	CCG AGG AGG AGG ACA AGA AG
		R	CTT CAG GAG TGA AGG CGA CA
CHOP	NM001195053	F	CTC CCA GAG CCC TCA CTC TC
		R	TGC TTG AGC CGT TCA TTC TC
VEGF	NM001171623	F	CTA CCT CCA CCA TGC CAA GT
		R	GCA GTA GCT GCG CTG ATA GA
GAPDH	NM002046	F	TTG GTA TCG TGG AAG GAC TC
		R	ACA GTC TTC TGG GTG GCA GT

### ELISA

VEGF protein levels in collected media were examined using a human VEGF ELISA Kit (R&D Systems, Minneapolis, MN, USA). Supernatant from incubated cells was collected and enzyme-linked immunosorbent assay (ELISA) was immediately performed, as described in the manufacturer’s protocol. We added 50 μL of assay diluent solution to the appropriate wells in the supplied microplate, followed by 200 μL of samples or standard. After 2 h of incubation, we aspirated and washed all wells and added 200 μL of substrate solution to each well. After another 20 min of incubation, 50 μL of stop solution was added to each well, and the optical density (OD) at 450 nm was immediately read. The OD of each sample was measured three times. The experiments were repeated at least three times and two or three samples were obtained from each independent experiment.

### Western blot analysis

Cells were lysed in ice-cold RIPA buffer [50 mM Tris-HCl (pH 8.0), 150 mM NaCl, 1% NP-40, 0.5% deoxycholate, and 0.1% SDS] for 30 min. Debris was removed by centrifugation at 16,000 *g* for 1 min. Equal amounts (20 μg) of total cell protein were separated by SDS-polyacrylamide gel electrophoresis (SDS-PAGE) and transferred to PVDF membranes. After blocking with 5% BSA in TTBS buffer (10 mM Tris, pH 8.0, 150 mM NaCl, 0.1% Tween 20) for 1 h at room temperature, membranes were incubated overnight at 4°C with the following primary antibodies: rabbit anti-BiP/GRP78 (1:1000; Cell Signaling, Beverly, MA, USA), mouse anti-GADD153 (CHOP; 1:200; Santa Cruz), and β-actin (1:10000; Sigma-Aldrich). The membranes were then incubated with a peroxidase-conjugated secondary antibody for 1 h at room temperature. Blots were developed using an enhanced chemiluminescence (ECL) kit (GE healthcare, Buckinghamshire, UK) and visualized using a Fujifilm Image Reader LAS-3000 (Fujifilm, Tokyo, Japan). Each experiment was repeated at least three times and the densitometric analysis was performed using a Multi Gauge V3.0 (Fujifilm Life Science, Tokyo, Japan).

### Statistical analyses

Data are presented as mean ± standard deviation (SD). Statistical analysis was performed using SPSS ver. 22.0 (SPSS Inc., Chicago, IL, USA). T-tests were performed to compare the differences between the two groups and a P value of less than 0.05 was considered statistically significant. One-way analysis of variance (ANOVA) followed by the Bonferroni correction of P values was performed for multiple comparisons and a P value of less than 0.01 was considered statistically significant.

## Results

### Cell viability

**Fig 1:**
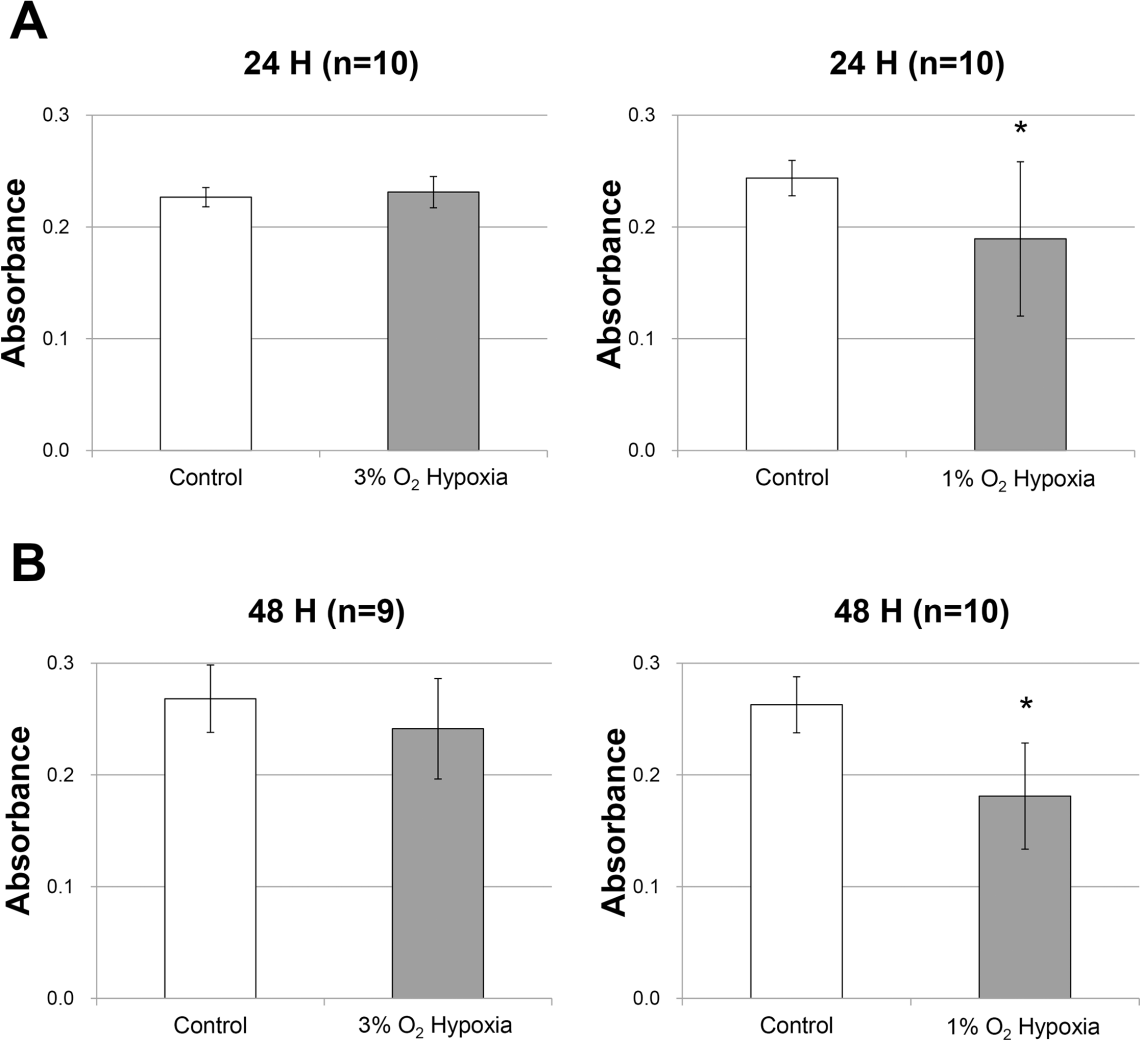
Effects of different oxygen concentrations on cell viability. RPE cells were incubated for 24 h and 48 h under normoxic conditions (control group), 3% O_2_ hypoxic conditions, or 1% O_2_ hypoxic conditions. Cell viability was measured by a PrestoBlue assay. The absorbance was read at 570 nm after RPE cells were incubated for 2 h with PB reagent. Corrected absorbance was calculated by subtracting the average control well value from that of each experimental well. **A.** No significant difference was observed between the control group and the 3% O_2_ hypoxic group (n = 10, p = 0.375), whereas the relative cell viability decreased to 77.7% after 24 h of incubation under 1% O_2_ hypoxic conditions compared with the control group (n = 10, p = 0.026). **B.** No significant difference was observed between the control group and the 3% O_2_ hypoxic group (n = 9, p = 0.142), whereas the relative cell viability decreased to 68.9% after 48 h of incubation under 1% O_2_ hypoxic conditions compared with the control group (n = 10, p < 0.001). Error bars represent ±1 standard deviation of the mean. P values were calculated by the t-test. An asterisk (*) indicates p < 0.05 versus control group.

In the preliminary experiments, no significant difference in cell viability was observed between the control group and the 3% O_2_ hypoxic group (p = 0.792 and p = 0.055, respectively), whereas the relative cell viability decreased to 77.7% and 68.9%, after 24 h and 48 h incubation, respectively, under 1% O_2_ hypoxic condition compared with that under 21% O_2_ normoxic condition (p = 0.023 and p < 0.001, respectively; Fig 1). Therefore, 1% O_2_ hypoxic condition was selected for the subsequent experiments.

**Fig 2:**
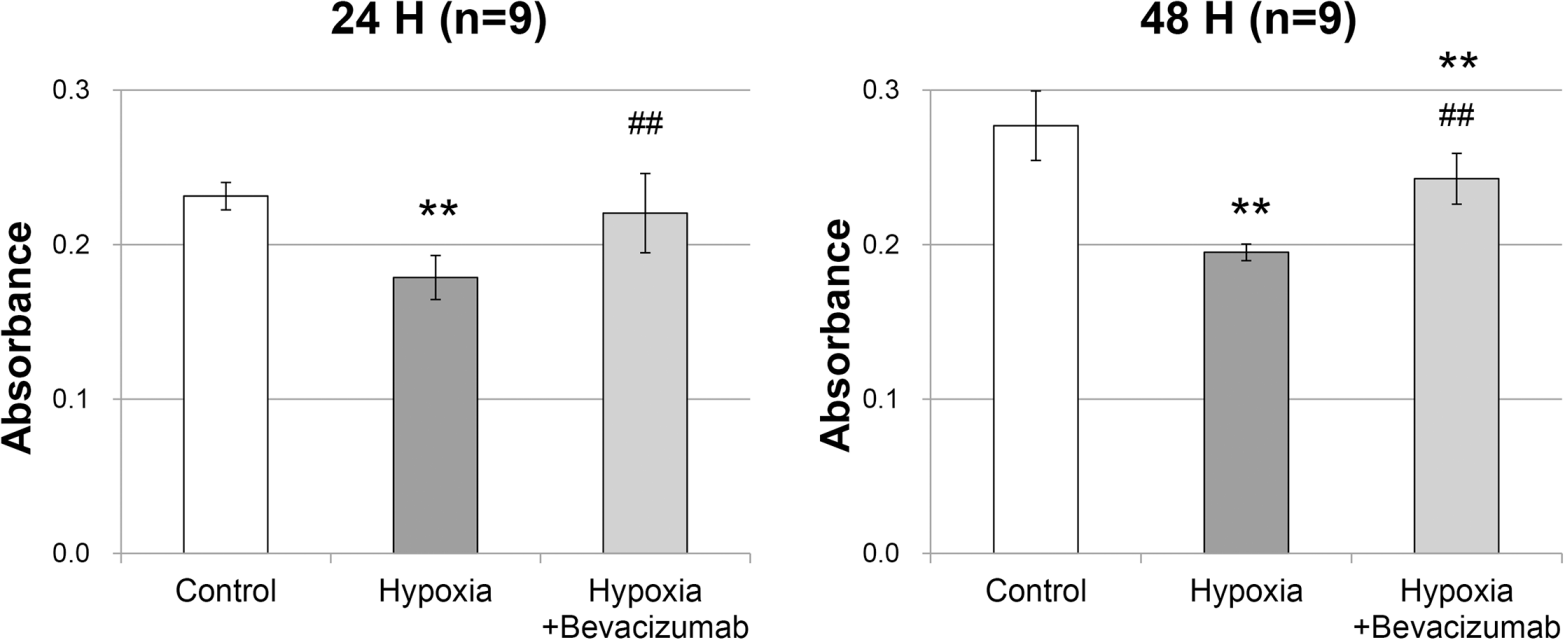
Cell viability measured by PrestoBlue assay. RPE cells were incubated for 24 h (n = 9) or 48 h (n = 9) under normoxic conditions (control group), 1% O_2_ hypoxic condition (hypoxia group), or 1% O_2_ hypoxic condition with 0.3125 mg/mL bevacizumab (hypoxia+bevacizumab group). Cell viability was measured by a PrestoBlue assay. The absorbance was read at 570 nm after RPE cells were incubated for 2 h with PB reagent. Corrected absorbance was calculated by subtracting the average control well value from that of each experimental well. Error bars represent ±1 standard deviation of the mean. Statistical significance was determined by one-way ANOVA followed by Bonferroni multiple comparison tests. Two asterisks (**) indicate p < 0.01 versus control group. Two hashtags (##) indicate p < 0.01 versus hypoxic group.

The mean relative cell viability decreased to 77.2% after 24 h of incubation and to 70.4% after 48 h of incubation under 1% O_2_ hypoxic condition compared with that under 21% O_2_ normoxic condition (p < 0.001 and p < 0.001, respectively; Fig 2). Under hypoxic condition, bevacizumab treatment improved the relative cell viability from 77.2% to 95.2% after 24 h of incubation and from 70.4% to 87.6% after 48 h of incubation (p = 0.001 and p < 0.001, respectively; Fig 2). There was no significant difference in cell viability between the control group and the hypoxia+bevacizumab group after 24 h of incubation (p = 0.170; Fig 2). After 48 h of incubation, cell viability was different between the control group and the hypoxia+bevacizumab group (p < 0.001; Fig 2).

**Fig 3:**
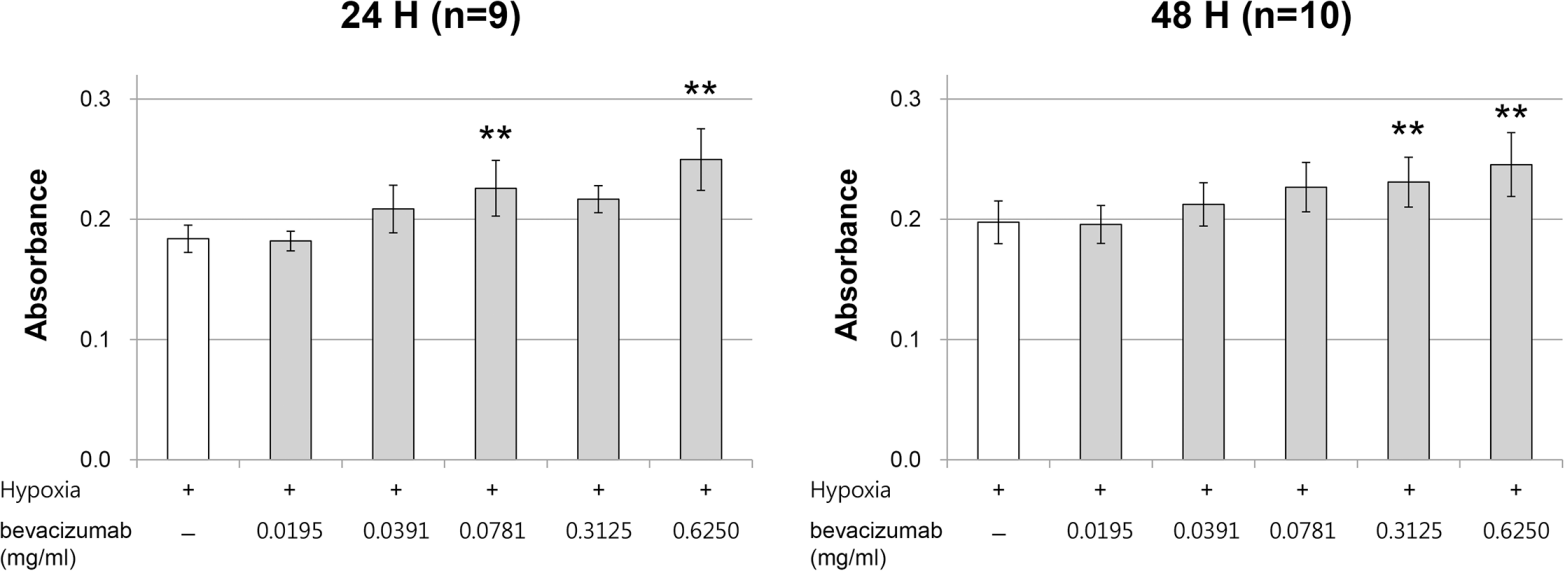
Effects of different concentrations of bevacizumab on cell viability of hypoxic RPE cells. RPE cells were incubated for 24 h (n = 9) and 48 h (n = 10) under 1% O_2_ hypoxic condition (hypoxia group) or 1% O_2_ hypoxic condition with final concentrations of 0.0195, 0.0391, 0.0781, 0.3125, or 0.6250 mg/mL of bevacizumab. Cell viability was measured by a PrestoBlue assay. The absorbance was read at 570 nm after RPE cells were incubated for 2 h with PB reagent. Corrected absorbance was calculated by subtracting the average control well value from that of each experimental well. The relative cell viability significantly increased to 122.8% and 135.8% after 24 h of incubation in groups treated with final concentrations of 0.0781 and 0.6250 mg/mL of bevacizumab, respectively, compared with that in the hypoxia group and to 116.9% and 124.3% after 48 h of incubation in groups treated with final concentrations of 0.3125 and 0.6250 mg/mL of bevacizumab, respectively, compared with that in the hypoxia group (p < 0.01). Error bars represent ±1 standard deviation of the mean. Statistical significance was determined by one-way ANOVA followed by Bonferroni multiple comparison tests. Two asterisks (**) indicate p < 0.01 versus hypoxia group.

The effects of different concentrations of bevacizumab on the cell viability of hypoxic RPE cells were investigated. The relative cell viability significantly increased to 122.8% and 135.8% after 24 h of incubation in groups treated with final concentrations of 0.0781 and 0.6250 mg/mL bevacizumab, respectively, compared with that in the hypoxia group (p < 0.001 and p < 0.001, respectively; Fig 3). Final concentrations of 0.0195, 0.0391, and 0.3125 mg/mL bevacizumab did not affect cell viability after 24 h of incubation (p = 0.999, p = 0.077, and p = 0.017, respectively). The relative cell viability significantly increased to 116.9% and 124.3% after 48 h of incubation in groups treated with a final concentration of 0.3125 and 0.6250 mg/mL bevacizumab, respectively, compared with that in the hypoxia group (p = 0.007 and p < 0.001, respectively; Fig 3). Final concentrations of 0.0195, 0.0391, and 0.0781 mg/mL bevacizumab did not affect cell viability after 48 h of incubation (p = 0.999, p = 0.999, and p = 0.031, respectively).

### ELISA for VEGF

**Fig 4:**
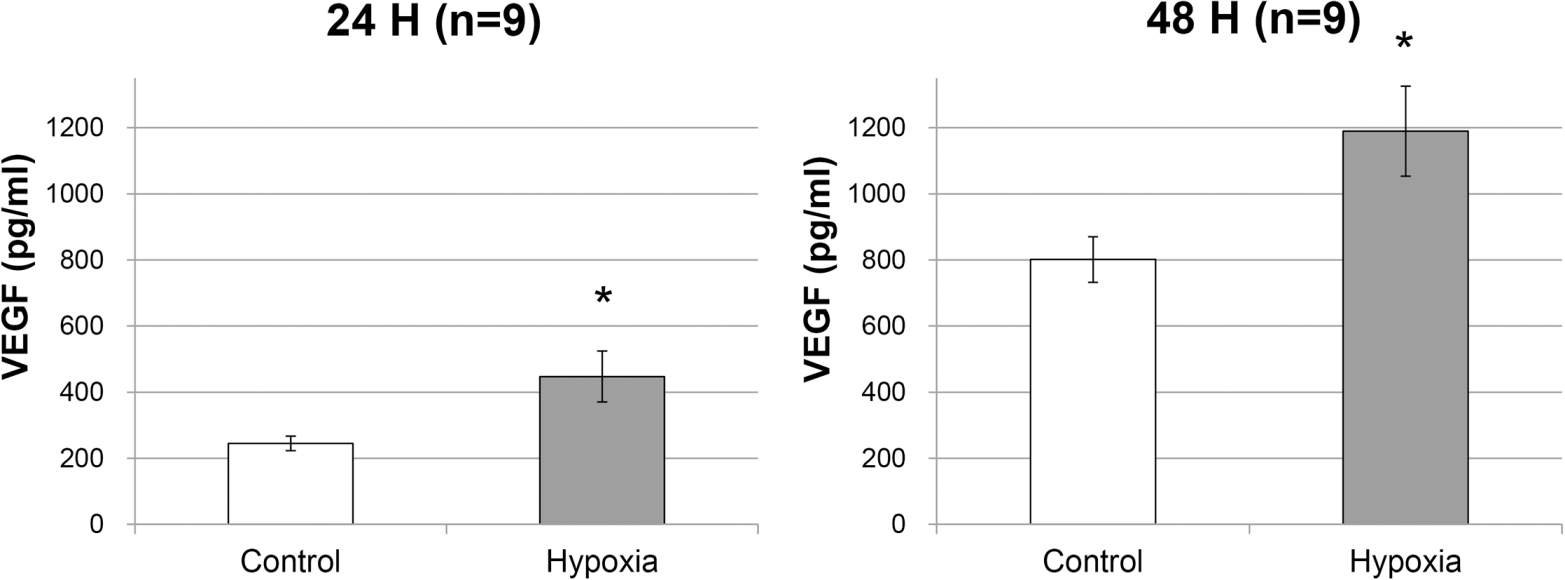
VEGF proteins in the culture media. RPE cells were incubated for 24 h (n = 9) or 48 h (n = 9) under normoxic condition (control group) or 1% O_2_ hypoxic condition (hypoxia group). Error bars represent ±1 standard deviation of the mean. Statistical significance was determined by the t-test. An asterisk (*) indicates p < 0.05 versus control group.

The mean concentration of VEGF in the media increased from 244.7 ± 21.82 pg/mL to 447.1 ± 76.97 pg/mL after 24 h of incubation and from 801.5 ± 69.11 pg/mL to 1189.8 ± 136.04 pg/mL after 48 h of incubation under hypoxic conditions compared with that under normoxic conditions (p < 0.001 and p < 0.001, respectively; Fig 4).

### Quantitative Real-Time PCR for VEGF, BiP/GRP78, and CHOP

**Fig 5:**
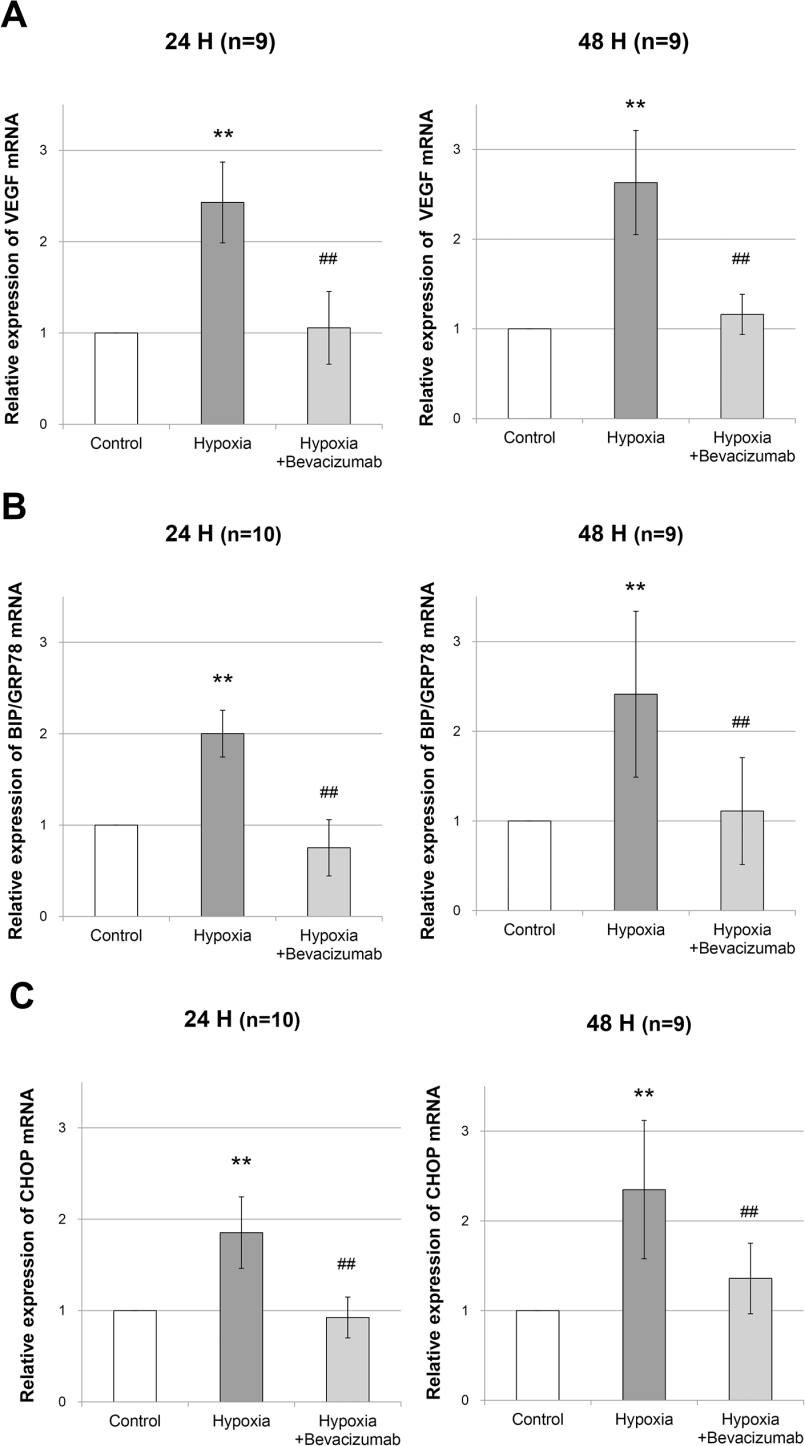
Quantitative real-time polymerase chain reaction (PCR) for VEGF, BiP/GRP78, and CHOP. RPE cells were incubated for 24 h or 48 h under normoxic conditions (control group), 1% O_2_ hypoxic condition (hypoxia group), or 1% O_2_ hypoxic condition with 0.3125 mg/mL bevacizumab treatment (hypoxia+bevacizumab group). Error bars represent ±1 standard deviation of the mean. Statistical significance was determined by one-way ANOVA followed by Bonferroni multiple comparison tests. Two asterisks (**) indicate p < 0.01 versus control group. Two hashtags (##) indicate p < 0.01 versus hypoxic group.

The relative level of VEGF mRNA increased 2.4 ± 0.44 fold after 24 h of incubation and 2.6 ± 0.58 fold after 48 h of incubation under hypoxic conditions compared that with under normoxic condition (p < 0.001 and p < 0.001, respectively; Fig 5A). Under hypoxic conditions, bevacizumab treatment resulted in a decreased level of VEGF mRNA from 2.4 ± 0.44 fold to 1.1 00B1 0.40 fold after 24 h of incubation and from 2.6 ± 0.58 fold to 1.2 ± 0.22 fold after 48 h of incubation (p < 0.001 and p < 0.001, respectively; Fig 5A). There were no significant differences in the levels of VEGF mRNA between the control group and the hypoxia+bevacizumab group after 24 h and 48 h of incubation (p = 0.999 and p = 0.999, respectively; Fig 5A).

The relative level of BiP/GRP78 mRNA increased 2.0 ± 0.26 fold after 24 h of incubation and 2.4 ± 0.93 fold after 48 h of incubation under hypoxic conditions compared with that under normoxic conditions (p < 0.001 and p < 0.001, respectively; Fig 5B). Under hypoxic conditions, bevacizumab treatment resulted in a decreased level of BiP/GRP78 mRNA from 2.0 ± 0.26 fold to 0.8 ± 0.31 fold after 24 h of incubation and from 2.4 ± 0.93 fold to 1.1 ± 0.60 fold after 48 h of incubation (p < 0.001 and p = 0.001, respectively; Fig 5B). There were no significant differences in the levels of BiP/GRP78 mRNA between the control group and the hypoxia+bevacizumab group after 24 h and 48 h of incubation (p = 0.069 and p = 0.999, respectively; Fig 5B).

The relative level of CHOP mRNA increased 1.9 ± 0.39 fold after 24 h of incubation and 2.3 ± 0.77 fold after 48 h of incubation under hypoxic conditions compared with that under normoxic conditions (p < 0.001 and p < 0.001, respectively; Fig 5C). Under hypoxic conditions, bevacizumab treatment caused a decrease in the level of CHOP mRNA from 1.9 ± 0.39 fold to 0.9 ± 0.22 fold after 24 h of incubation and from 2.3 ± 0.77 fold to 1.4 ± 0.39 fold after 48 h of incubation (p < 0.001 and p = 0.001, respectively; Fig 5C). There were no significant differences in the levels of CHOP mRNA between the control group and the hypoxia+bevacizumab group after 24 h and 48 h of incubation (p = 0.999 and p = 0.425, respectively; Fig 5C).

### Western blot analysis for BiP/GRP78 and CHOP

**Fig 6:**
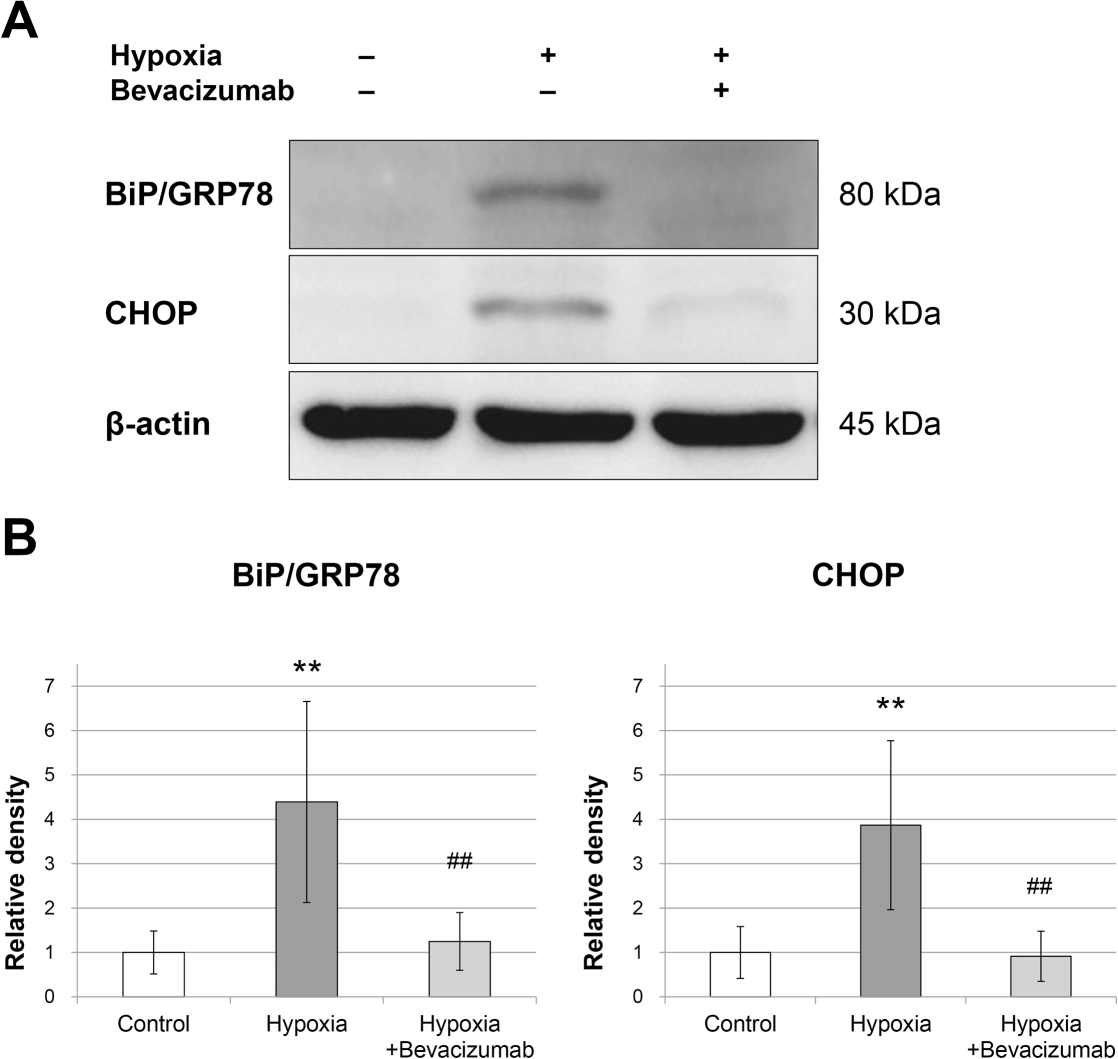
Western blot analyses of BiP/GRP78 and CHOP. RPE cells were incubated for 48 h under normoxic conditions (control group), 1% O_2_ hypoxic condition (hypoxia group), or 1% O_2_ hypoxic condition with 0.3125 mg/mL bevacizumab treatment (hypoxia+bevacizumab group). Protein levels were quantified by normalization to β-actin level (B). Error bars represent ±1 standard deviation of the mean. Statistical significance was determined by one-way ANOVA followed by Bonferroni multiple comparison tests. Two asterisks (**) indicate p < 0.01 versus control group. Two hashtags (##) indicate p < 0.01 versus hypoxic group.

The protein levels of both BiP/GRP78 and CHOP increased after 48 h of incubation under hypoxic conditions compared with that under normoxic conditions (p < 0.001; Fig 6). Under hypoxic conditions, bevacizumab treatment resulted in decreased protein levels of BiP/GRP78 and CHOP (p < 0.001 and p < 0.001, respectively; Fig 6). There were no significant differences in the protein levels of both BiP/GRP78 and CHOP between the control group and the hypoxia+bevacizumab group after 48 h of incubation (p = 0.999 and p = 0.999, respectively; Fig 6).

## Discussion

Cells under low oxygen tension adapt to maintain homeostasis. However, if this fails, the cells die by apoptosis [21]. One cellular response to low oxygen is the stabilization of hypoxia-inducible factor-1 (HIF-1), a transcription factor that upregulates VEGF expression [22–24]. VEGF is a major factor in the development of angiogenesis that occurs in wet AMD [22]. Previous studies suggested that because of decreased choroidal circulation, ischemia and hypoxia may play a role in the development of CNV in AMD [25, 26]. In the present study, we showed that VEGF secretion into media increased in RPE cells cultured under hypoxic conditions for 24 and 48 h.

The induction of ER stress and UPR is another cellular response to low oxygen [27]. Although the precise mechanism of how unfolded or misfolded proteins accumulate in the ER under low oxygen tension remains to be elucidated, one possible explanation is that molecular oxygen is the major electron acceptor providing the driving force for protein folding in the ER [28]. The UPR improves protein folding by the upregulation of ER chaperones, promotes unfolded protein degradation by promoting ER-associated degradation (ERAD), and reduces the number of new proteins entering the ER by slowing protein translation [29]. These efforts can eliminate ER stress. However, in the face of prolonged ER stress, apoptotic cell death is triggered by the release of calcium from the ER and the stimulation of CHOP expression, which also enhances apoptosis [30, 31]. ER stress and the UPR are involved in the RPE apoptosis induced by cigarette smoke-related oxidative injury [17]. In the present study, we showed that BiP/GRP78 and CHOP, which are the two main proteins involved in ER stress and the UPR, increased and cell viability decreased in hypoxic RPE cells. ER stress induced by hypoxia may be also implicated in the pathogenesis of AMD.

In this study, the increases in BiP/GRP78 and CHOP mRNA were small in the hypoxia group compared with those in the control group. In a study using astrocytes, CHOP mRNA levels reached values almost 70 times higher than those of controls 6 h after oxygen and glucose deprivation [32]. This study, however, used a different type of cells and different conditions. In addition, we also determined the changes in BiP/GRP78 and CHOP protein levels in the hypoxia group after 48 h of incubation. These changes were similar to changes in their mRNA levels.

Bevacizumab is a humanized monoclonal antibody against human VEGF-A isoforms [33]. Although other anti-VEGF drugs exist, such as ranibizumab and aflibercept that have been approved by the Food and Drug Administration (FDA) for the treatment of wet AMD, many ophthalmologists are using off-label bevacizumab because of its low cost [7]. Anti-VEGF drugs are effective for treating wet AMD, but in most cases, many injections are required [7]. However, VEGF plays an important role in the survival and maintenance of RPE cell integrity [34], and RPE-derived VEGF is essential for the maintenance of choriocapillaries [35]. In addition, VEGF has been reported to be involved in neuroprotection of the retina [36]. Recent reports suggested that RPE atrophy and choroidal atrophy were exacerbated by anti-VEGF treatments for wet AMD [37–39]. Increased RPE atrophy in the macula has been correlated with a poor visual outcome [40].

In the present study, we induced ER stress by exposing RPE cells to hypoxic conditions and investigated the effects of bevacizumab, an anti-VEGF drug, on ER stress. Our results indicated that bevacizumab treatment mitigated ER stress in human RPE cells cultured under hypoxic conditions. Under hypoxic conditions, bevacizumab treatment decreased the expression of both BiP/GRP78 and CHOP mRNA and protein. However, we cannot elucidate the mechanism for how bevacizumab affects ER stress. One possible explanation is that the paracrine effects of secreted VEGF between RPE cells were blocked by bevacizumab [23]. Karali et al. [41] demonstrated in an *in vitro* study using endothelial cells that VEGF activated UPR mediators without the accumulation of unfolded proteins in the ER, and suggested that the activation of these mediators, activating transcription factor 6 (ATF6) and PKR-like endoplasmic reticulum kinase (PERK), contributed to the survival effect of VEGF on endothelial cells.

In addition, this study demonstrated that bevacizumab treatment improved cell viability and reduced the expression of VEGF mRNA under hypoxic conditions. The decreased expression of CHOP, which is closely linked to apoptosis, after bevacizumab treatment may be associated with improved cell viability. Reduced ER stress, which is an inducer of VEGF expression, after bevacizumab treatment may explain the reduced expression of VEGF mRNA. ER stress is an HIF-1-independent inducer of VEGF and may contribute to CNV formation in AMD [18, 19, 42]. Studies in which different ER stress models were used revealed that the expression of VEGF was activated via ATF4 in RPE cells [43, 44].

In the present study, we investigated the effects of various concentrations of bevacizumab on the viability of hypoxic RPE cells. Cell viability significantly increased in groups treated with final concentrations of 0.0781 and 0.625 mg/mL bevacizumab after 24 h of incubation and final concentrations of 0.3125 and 0.625 mg/mL bevacizumab after 48 h of incubation compared with that in the hypoxia group. Low concentrations of bevacizumab did not affect cell viability. A final concentration of 0.3125 mg/mL bevacizumab is the approximate theoretical concentration immediately following injection of this drug into human eyes [20]. The pharmacokinetics of intravitreal bevacizumab should be considered clinically. In the rabbit eye, following intravitreal injection of 1.25 mg of bevacizumab, the peak concentration in vitreous humor was 0.4 mg/mL after 1 day and decreased in a single exponential fashion afterwards with a half-life of 4.32 days [45]. In patients with CNV, the pharmacokinetics of intravitreal bevacizumab followed a two-compartment model with initial and terminal half-lives of 0.5 and 6.7 days, respectively [46]. The peak concentration (0.165 mg/mL) was reached on day 2 after the intravitreal injection of 1.25 mg of bevacizumab and the estimated value was 0.053 mg/mL on day 4 [46]. In addition, it should be considered that the concentration of bevacizumab may be different between the vitreous and RPE. Therefore, the effects of bevacizumab on RPE cells under the experimental conditions are unlikely to be clinically equivalent to those in the human eye.

Since this study conducted *in vitro* using cells, the clinical effect of bevacizumab should be judged carefully. Bevacizumab may induce atrophy of choriocapillaries, which is subsequently associated with RPE atrophy [35]. Therefore, further *in vivo* experiments are needed to confirm our results. In addition, we exposed RPE cells to only hypoxic conditions in this study. Oxidative stress is another important factor implicated in the pathogenesis of AMD [47]. In previous *in vitro* experiments, bevacizumab treatment aggravated cell death under high oxidative stress conditions [48, 49]. Another limitation in the present study was that we did not investigate the effect of other anti-VEGF drugs, such as ranibizumab and aflibercept, on ER stress. Malik et al. [20] reported different effects of anti-VEGF drugs on mitochondrial toxicity.

In conclusion, this *in vitro* study demonstrated that bevacizumab mitigated ER stress in human RPE cells cultured under hypoxic conditions. This effect may be involved in improved cell viability and the reduction of VEGF expression by bevacizumab in hypoxic RPE cells *in vitro*. However, the effects of bevacizumab on RPE cells under experimental conditions are unlikely to be clinically equivalent to those in the human eye. Further studies on the mechanism of bevacizumab affecting ER stress are necessary.
